# Supplementary material for: Mapping dopaminergic projections in the human brain with resting-state fMRI
Source: eLife. 2022 Feb 3;11:e71846. doi: 10.7554/eLife.71846 (PMC8843090; doi:10.7554/eLife.71846)
Supplement: Transparent reporting form [file elife-71846-transrepform1.docx]

***eLife’s* transparent reporting form**

We encourage authors to provide detailed information *within their submission* to facilitate the interpretation and replication of experiments. Authors can upload supporting documentation to indicate the use of appropriate reporting guidelines for health-related research (see [EQUATOR Network](http://www.equator-network.org/%20)), life science research (see the [BioSharing Information Resource](https://biosharing.org/)), or the [ARRIVE guidelines](http://www.plosbiology.org/article/info:doi/10.1371/journal.pbio.1000412) for reporting work involving animal research. Where applicable, authors should refer to any relevant reporting standards documents in this form.

If you have any questions, please consult our Journal Policies and/or contact us: [editorial@elifesciences.org](mailto:editorial@elifesciences.org).

**Sample-size estimation**

- You should state whether an appropriate sample size was computed when the study was being designed
- You should state the statistical method of sample size computation and any required assumptions
- If no explicit power analysis was used, you should describe how you decided what sample (replicate) size (number) to use

We made use of two publicly available datasets, and one locally acquired dataset. For all of these datasets, the data had already been acquired for the purpose of other experiments, prior to the start of our study, so no specific sample size calculations were performed for our current analyses. However, for all of our analyses, we always included as many subjects as possible. The number of subjects included in each of our analyses is listed below. As can be observed, these numbers are either comparable to subject numbers typically reported in the literature (local PD dataset & HCP substance use analysis), or they are substantially higher.

1) HCP dataset main analysis:	839 subjects

2) PPMI dataset DaT SPECT:	209 control subjects

3) PPMI dataset within-subject resting-state fMRI & DaT SPECT:	144 subjects

4) Local Parkinson's disease (PD) dataset:	20 healthy controls, 39 PD patients

5) HCP substance use analyses:	30 alcohol and 38 nicotine users

These subject numbers and more information can be found in following sections of our manuscript:

# 1) Materials and Methods *–* Resting-state fMRI data of the Human Connectome Project dataset

2) Materials and Methods ***–*** DaT SPECT imaging in the PPMI dataset

3) Materials and Methods ***–*** Mapping the second-order striatal connectivity mode onto DaT availability

4) Materials and Methods ***–*** Resting-state fMRI data of the Parkinson’s disease dataset (and Table 2)

5) Materials and Methods ***–*** Investigating the second-order striatal mode in relation to tobacco and alcohol use

Please outline where this information can be found within the submission (e.g., sections or figure legends), or explain why this information doesn’t apply to your submission:

**Replicates**

- You should report how often each experiment was performed
- You should include a definition of biological versus technical replication
- The data obtained should be provided and sufficient information should be provided to indicate the number of independent biological and/or technical replicates
- If you encountered any outliers, you should describe how these were handled
- Criteria for exclusion/inclusion of data should be clearly stated
- High-throughput sequence data should be uploaded before submission, with a private link for reviewers provided (these are available from both GEO and ArrayExpress)

Our manuscript describes a comprehensive series of experiments, consisting out of a total of 6 different analyses using data from three different datasets to demonstrate that our resting-state fMRI derived striatal marker can be used as a biomarker for dopaminergic functioning. As such, a typical, out of sample replication of all these analyses, would be beyond the scope of the current manuscript. However, we did conduct multiple (within-sample) analyses demonstrating the validity of our results. See below for a summary of these analyses and the respective sections of the manuscript that contain more detailed information.

HCP dataset – Analyses of reproducibility second order connectivity mode:

We demonstrate that the subject-specific resting-state fMRI derived connectivity modes are highly consistent across the two fMRI sessions. We also demonstrated that the Inter-class correlation (ICC(2,k)), which indexes validity for a putative biomarker showed excellent reproducibility of the subject-specific connectivity modes while still being sensitive to inter-individual differences.

# See for more detailed information:

# *Materials and Methods – Resting-state fMRI data of the Human Connectome Project dataset*

*Results – Striatal connection topographies map onto DaT availability (3^rd^ paragraph)*

PPMI dataset – Analyses of correspondence DaT-SPECT & second order connectivity mode:

We demonstrate that the group-level correspondence between the resting-state fMRI derived connectivity mode and the DaT SPECT scan can also be replicated at the single subject level.

See for more detailed information:

*Materials and Methods* ***–*** *Mapping the second-order striatal connectivity mode onto DaT availability*

# *Appendix 1 - Supplementary Analyses: Within-subject correspondence between the second-order striatal connectivity mode and the DaT SPECT scan*

Please outline where this information can be found within the submission (e.g., sections or figure legends), or explain why this information doesn’t apply to your submission:

Local PD dataset – Analyses of effects of diagnosis and L-DOPA administration:

We demonstrate that the PD-related alterations and the effects of L-DOPA are independent of age and gender.

# See for more detailed information:

# *Materials and Methods – Resting-state fMRI data of the Parkinson’s Disease dataset*

# *Appendix 1 - Supplementary Analyses: Post-hoc analyses of age and sex*

HCP dataset - Substance use analyses:

We conducted post-hoc analyses demonstrating that our results could be replicated under different usage thresholds and were independent of age and gender.

See for more detailed information:

*Materials and Methods – Investigating the second-order striatal mode in relation to tobacco and alcohol use*

# *Appendix 1 - Supplementary Analyses: Post-hoc analyses of age and sex*

# *Appendix 1 - Supplementary Analyses: Post-hoc analyses using different usage thresholds for tobacco and alcohol use*

**Statistical reporting**

- Statistical analysis methods should be described and justified
- Raw data should be presented in figures whenever informative to do so (typically when N per group is less than 10)
- For each experiment, you should identify the statistical tests used, exact values of N, definitions of center, methods of multiple test correction, and dispersion and precision measures (e.g., mean, median, SD, SEM, confidence intervals; and, for the major substantive results, a measure of effect size (e.g., Pearson's r, Cohen's d)
- Report exact p-values wherever possible alongside the summary statistics and 95% confidence intervals. These should be reported for all key questions and not only when the p-value is less than 0.05.

Please outline where this information can be found within the submission (e.g., sections or figure legends), or explain why this information doesn’t apply to your submission:

HCP dataset – Analyses of reproducibility second order connectivity mode:

# - Materials and Methods – Connectopic mapping of the striatum in the Human Connectome Project dataset

# - Results – Striatal connection topographies map onto DaT availability

- Results – Table 1

# - Appendix 1 - Supplementary Methods: Post-hoc analyses comparing the second-order striatal connectivity mode with PET markers of other neurotransmitter systems

# - Figure 2-figure supplement 1-3

Analyses of correspondence DaT-SPECT & second order connectivity mode:

- Materials and Methods **–** Mapping the second-order striatal connectivity mode onto DaT availability

- Results – Striatal connection topographies map onto DaT availability

- Results – Figures 2 and 3

# - Appendix 1 - Supplementary Methods: Within-subject correspondence between the second-order striatal connectivity mode and the DaT SPECT scan

# - Figure 3-figure supplement 1 and 2

PD dataset – Analyses of effects of diagnosis and L-DOPA administration:

# - Materials and Methods – Investigating the second-order striatal mode in Parkinson’s Disease dataset

- Results – Striatal connection topographies are altered in Parkinson Disease

- Results – Striatal connection topographies are sensitive to acute dopaminergic modulation

- Results – Figures 4 and 5

# - Appendix 1 - Supplementary Methods: Post-hoc analyses of age and sex

# - Appendix 2 - Table 1

HCP dataset – Substance use analyses:

- Materials and Methods – Investigating the second-order striatal mode in relation to tobacco and alcohol use

- Results – Striatal connection topographies are associated with the amount of substance use

- Results – Figure 6

# - Appendix 1 - Supplementary Methods: Post-hoc analyses of age and sex

# - Appendix 1 - Supplementary Methods: Post-hoc analyses using different usage thresholds for tobacco and alcohol use

- Figure 6-figure supplement 1 and 2

- Appendix 2-Tables 1-3

(For large datasets, or papers with a very large number of statistical tests, you may upload a single table file with tests, Ns, etc., with reference to sections in the manuscript.)

**Group allocation**

- Indicate how samples were allocated into experimental groups (in the case of clinical studies, please specify allocation to treatment method); if randomization was used, please also state if restricted randomization was applied
- Indicate if masking was used during group allocation, data collection and/or data analysis

Please outline where this information can be found within the submission (e.g., sections or figure legends), or explain why this information doesn’t apply to your submission:

HCP dataset:

Not applicable, no experimental manipulation/group allocation, participants just underwent 1 or 2 resting-state fMRI sessions.

PPMI dataset:

Not applicable, no experimental manipulation/group allocation, participants just underwent DAT SPECT scan (and a subsample also underwent a resting-state fMRI scan).

Local PD dataset:

The order of the placebo session and L-DOPA session was randomized, and allocation to the placebo session and L-DOPA session was double-blind during data-collection, see the paragraph “Resting-state fMRI data of the Parkinson’s disease dataset” in the Materials and Methods section for more detailed information.

**Additional data files (“source data”)**

- We encourage you to upload relevant additional data files, such as numerical data that are represented as a graph in a figure, or as a summary table
- Where provided, these should be in the most useful format, and they can be uploaded as “Source data” files linked to a main figure or table
- Include model definition files including the full list of parameters used
- Include code used for data analysis (e.g., R, MatLab)
- Avoid stating that data files are “available upon request”

Please indicate the figures or tables for which source data files have been provided:

The source data of Table 2 can be found in the Table2_Source_Data1.xlsx file.

All code used for the connectopic mapping procedure is available at the following Github repository: <https://github.com/koenhaak/congrads>.

The subject identifiers from the HCP dataset used in our analyses (Figure 2) are listed in Appendix 2 - Table 4. The subject identifiers from the PPMI dataset used in our analyses (Figures 2 and 3) are listed in Appendix 2 - Tables 5 and 6.
